# Social isolation during adolescence causes increased generalised anxiety-like behaviour in male rats and increased sociability in male and female rats

**DOI:** 10.1016/j.nsa.2024.104068

**Published:** 2024-04-14

**Authors:** Marina Manojlović, Filip Milosavljević, Andrea Atanasov, Bojan Batinić, Pavle Sitarica, Vesna Pešić, Marin M. Jukić

**Affiliations:** a Department of Physiology, Faculty of Pharmacy, University of Belgrade, Serbia; b Department of Psychiatry and Psychotherapy, School of Medicine, Technische Universität München, Germany; c Section of Pharmacogenetics, Department of Physiology and Pharmacology, Karolinska Institutet, Stockholm, Sweden

**Keywords:** Meta-analysis, Animal model, Open field, Elevated plus maze, Three-chamber test

## Abstract

As one of the main characteristics of adolescence is the increased desire for social interaction and formation of meaningful relationships, it represents a particularly vulnerable period for social stressors. Moreover, social isolation during adolescence can significantly alter brain function and increase the likelihood of anxiety disorders in early adulthood. The aim of this retrospective meta-analysis and prospective behavioural experiment was to investigate whether chronic isolation stress during adolescence causes generalised and social anxiety-like behaviour in rodents. The meta-analysis of previously published data showed that social isolation during adolescence leads to an increase in generalised anxiety-like behaviour exclusively in male isolated rodents and has ambiguous effect on social anxiety-like behaviour. In the subsequent prospective experiment, Sprague-Dawley rats of both sexes were subjected to six weeks of social isolation and their behaviour was investigated in the open field, elevated plus maze, and three-chamber test. Consistent with the retrospective literature analysis, social isolation in adolescence increased generalised anxiety-like behaviour exclusively in males, as indicated by a decrease in time spent in the centre of the open field and open arms of the elevated plus maze, compared to controls. Both male and female isolated rats showed an increase in sociability in the three-chamber test, as indicated by the increase in the preference ratio for social versus neutral stimuli, compared to controls. Altogether, social isolation leads to an increase in generalised anxiety-like behaviour exclusively in males and to increased sociability in both males and females.

## 1 Introduction

Anxiety disorders are characterised by fear or avoidance of a range of external and internal stimuli and can manifest in various forms, such as highly prevalent generalised anxiety disorder and social anxiety (Craske and Stein, 2016). It is estimated that the prevalence of anxiety disorders has increased by around 25% as a result of the COVID-19 pandemic, which is probably at least partly due to social isolation (Collaborators, 2021). Although social isolation can lead to maladaptive phenotypes in all circumstances, interaction with peers is particularly important for adolescents as this is a time when brain plasticity is particularly pronounced as social skills are developed and meaningful lifelong social relationships are formed. (Sawyer et al., 2012). Even apart from the COVID-19 pandemic, social isolation among adolescents is increasingly common in today's world due to social media, video games and increasing social pressure. Many findings suggest that social isolation during this time can cause structural, functional and behavioural changes in the brain that can significantly increase the likelihood of developing anxiety disorders in early adulthood (Lukkes et al., 2009).

As sociability is an evolutionarily conserved trait in mammals (Douglas et al., 2004), social isolation stress during adolescence in rodents has often been used as a model to mechanistically understand the relationship between social isolation in adolescence and the risk of anxiety disorders (Lezak et al., 2017). However, despite considerable effort, studies conducted to date have been inconclusive about the behavioural outcomes of social isolation stress in rodents, as results often differ depending on the experimental protocol, species, behavioural tests and sex of the animals. Furthermore, most studies on social isolation in adolescence focus exclusively on males, although females are almost twice as likely to be affected by anxiety disorders (Scholl et al., 2019). The aim of this retrospective meta-analysis and prospective behavioural experiment was therefore to investigate whether chronic isolation stress in adolescence induces generalised and social anxiety-like behaviour in male and female rats.

## 2 Methods

### 2.1 Literature survey

To investigate the effects of social isolation during adolescence in rodents, a literature survey was conducted and the results quantified by meta-analysis. Inclusion criteria were: (1) study was performed on rodents, (2) animals were isolated during the adolescence between postnatal day 21 and 90 for at least 2 weeks, (3) tests for generalised anxiety-like behaviour: (*i*) open field test (OF), (*ii*) elevated plus maze test (EPM), or (*iii*) light-dark box test (LDB) or for social anxiety-like behaviour: (*i*) three-chamber test (3CT), (*ii*) social interaction test, or (*iii*) social preference test (SPT). Exclusion criteria were: (1) daily short-term isolation during adolescence accompanied by re-housing with cage mates, (2) prolonged isolation over 10 weeks, (3) confounding factor of chronic variable social stress and social defeat paradigms present, (4) surgery, invasive treatment, or any other substantial stress that could affect the animals' behaviour, (5) re-socialization prior to the behavioural test. If more than one test was used in studies to assess generalised or social anxiety, results were extracted from only one test in the following order: OF > EPM > LDB; and 3CT > SPT > SIT. Parameters used to approximate and quantify generalised and social anxiety-like behaviours were: (1) time spent in the centre of the OF (s/min or %), (2) time spent in the open arms in the EPM (s/min or %), (3) time spent in the light in LDB (s/min or %), (4) social interaction time (s/min or %), (5) social preference index, (6) interaction zone time (s/min). The mean values and standard deviations were extracted from manuscript if such results were available or from the graphs using established procedures of data transformation (Higgins et al., 2023).

### 2.2 Experimental animals

Three-week old male and female Sprague-Dawley rats were obtained from the Sprague-Dawley colony of the Faculty of Pharmacy - University of Belgrade, Serbia. Animals were kept under standard laboratory conditions that included 12 h light-dark cycle, controlled room temperature (21 ± 2 °C), humidity (40–45 %) and illumination (120 lx). The study procedures were approved by the Ethical Committee on Animal Experimentation of the Faculty of Pharmacy - University of Belgrade and Ministry of Agriculture, Forestry and Water Management of the Republic of Serbia - Veterinary Directorate of the Republic of Serbia (323-07-04232/2022–05).

### 2.3 Experimental design

In the fourth postnatal week, male and female rats were randomly assigned to the socially isolated or group-housed conditions. The socially isolated rats, which were used as experimental group, were single caged and had no direct contact with other rats, but could smell and hear them. Socially isolated rats were neither disturbed nor handled, except once a week while changing the bedding. Group-housed rats were maintained in groups of four in standard cages, handled three times per week, and were used as а control group free of social isolation stress (Fig. 2A).

### 2.4 Behavioural testing

Behavioural test battery was performed at postnatal week 10, with at least two days between each individual test. One week prior to testing, all rats were handled daily to reduce disrupting manipulation stress. Animals were allowed to acclimate to the experimental room for at least 30 min prior to testing. The socially isolated rats were kept in isolation for the entire duration of the experiment. All behavioural tests took place during the light phase of the cycle under indirect, dim light in the experimental room. ANY-maze software (Stoelting Co., Wood Dale, IL, USA) was used to track and analyse animal behaviour. The open field and elevated plus maze tests were used to assess generalised anxiety-like behaviour, while the three-chamber test was used to assess social anxiety-like behaviour.

#### 2.4.1 Open field test

The open field test was performed in a black plastic arena (120 × 120 × 50 cm). Rats were placed in the centre of the open field and observed for 15 min. The time spent in the centre was measured and decrease in this parameter was interpreted as an increase in generalised anxiety-like behaviour, while the total distance travelled in the open field was measured to quantify animal mobility.

#### 2.4.2 Elevated plus maze test

The elevated plus maze test was performed as previously described (Javorac et al., 2022) in a black plastic maze with two opposing open arms (50 × 10 cm) and two opposing closed arms (50 × 10 × 40 cm) connected by a central platform (10 × 10 cm). Rats were placed on the central of the platform with their heads facing one of the closed arms and were observed for 5 min. The time in the open arms (T_OA_), time in the closed arms (T_CA_), number of entries to the open arms (E_OA_) and number of entries to the closed arms (E_CA_) were measured. Subsequently, the time ratio (T_OA_/(T_OA_ + T_CA_)) and entry ratio (E_OA_/(E_OA_ + E_CA_)) were calculated and decrease in these parameters was interpreted as an increase in anxiety-like behaviour.

#### 2.4.3 Three-chamber test

The three-chamber test was performed in a black plastic apparatus (120 × 40 × 50 cm) consisting of three equal chambers (40 × 40 × 50 cm) connected by two plastic doors. The left and right chambers contained cylindrical cages that allowed close interaction between the test and stimulus rats. The three-chamber test consisted of three phases: habituation, social preference and social novelty. In the habituation phase, the test rat was placed in the central chamber with the doors open and allowed to freely explore the apparatus and empty cages for 5 min. In the social preference phase, the test rat was gently guided into the central chamber and the doors were closed while the stimulus rat was placed in one of the cages. The doors were opened, and the test rat was allowed to explore for 10 min. The time the rat spent with the social stimulus (T_SS_) and the empty cage as the non-social stimulus (T_NS_) was measured. The social preference index (SPI) was calculated as (T_SS_ − T_NS_)/(T_SS_ + T_NS_), in accordance with previously established method (Rein et al., 2020), and a decrease in this parameters was interpreted as an increase in social anxiety-like behaviour. During the social novelty phase, the test rat was again gently guided into the central chamber and the doors were closed. The first stimulus rat remained in the same cage while the second rat was placed in the other cage. The doors were opened and the test rat was allowed to explore for 10 min. The time the rat spent with the familiar rat (T_F_) and with the novel rat (T_N_) was measured. The social novelty index (SNI) was calculated as (T_N_ – T_F_)/(T_N_ + T_F_), in accordance with previously established method (Rein et al., 2020), and an increase in this parameter was interpreted as an increase in sociability. During the test, the social stimuli rats were kept in cages so that they could not fully express their social behaviour and possible aggression towards the test rats. The rats that were used as social stimuli were the same sex, age and strain as the test rats and were kept in different cages so that they were not known to the test rats. The position of the stimuli rats in the two side chambers was counterbalanced between tests.

### 2.5 Statistical analysis

The statistical analyses of the literature survey were performed by meta-analysis, using RevMan software, version 5.4 (Cochrane). Due to the large heterogeneity between the studies, a random effects model was used and the results were presented as the standardized mean differences.

The statistical analysis of the social isolation experiment was performed using the IBM SPSS Statistics 20 program. All graphs were created using GraphPad Prism 8.0.1 software. Data distribution was assessed using the Shapiro-Wilk test. Normally distributed data were analysed by two-way analysis of variance (ANOVA), with social isolation and animal sex as factors, and followed up by LSD post-hoc tests, if applicable. Data that didn't show a normal distribution were analysed with the Kruskal-Wallis test and followed up by Mann-Witney post hoc tests, if applicable. The p-values between groups lower than 0.05 were interpreted as statistically significant difference, while the values higher than 0.1 were interpreted as the absence of statistically significant difference between groups in the particular experiment. Confidence intervals for comparison of isolated to control animals related to social preference- and social novelty-related parameters were calculated by using the pooled values for males and females. Differences between groups were presented as means with a 95% confidence interval for normally distributed data or as median and IQR for non-normally distributed data.

## 3 Results

**Fig. 1:**
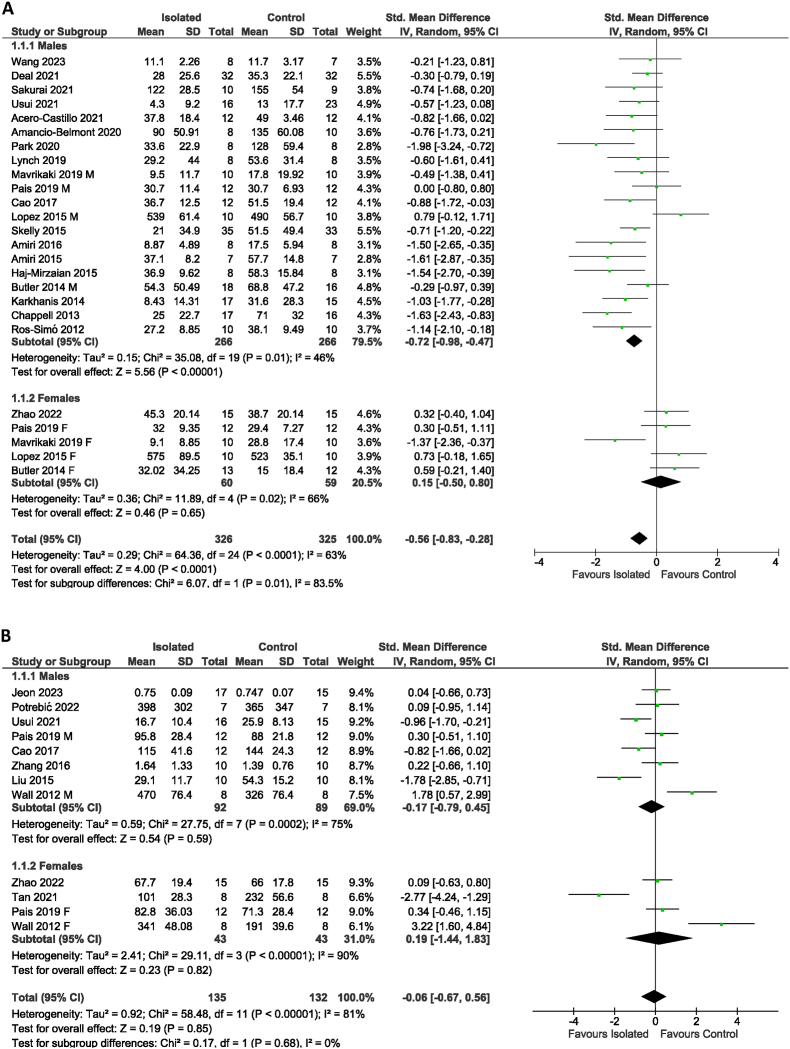
**Meta-analysis of effect of social isolation during adolescence** on (A) generalised and (B) social anxiety-like behaviour in male and female rodents.

Of the 176 studies initially screened for the inclusion, 28 studies (Park et al., 2020; Karkhanis et al., 2014; Liu et al., 2015; Mavrikaki et al., 2019; Zhao et al., 2022; Wang et al., 2022; Pais et al., 2019; Amiri et al., 2015, 2016; Tan et al., 2021; Amancio-Belmont et al., 2020; Wall et al., 2012; Potrebic et al., 2022; Butler et al., 2014a, 2014b; Lopez and Laber, 2015; Sakurai et al., 2021; Ros-Simo and Valverde, 2012; Deal et al., 2021; Usui et al., 2021; Cao et al., 2017; Haj-Mirzaian et al., 2015; Acero-Castillo et al., 2021; Skelly et al., 2015; Zhang et al., 2016; Chappell et al., 2013; Jeon et al., 2023; Lynch et al., 2019) were included in the meta-analyses for generalised or social anxiety-like behaviour. According to the pooled data from 21 studies, socially isolated male animals exhibited an increase in generalised anxiety-like behaviour (Standard mean difference: −0.72) compared to the male control animals (*p* < 0.001, Fig. 1A), while there was no statistically significant between-group difference in female animals (*p* > 0.1, Fig. 1A). The meta-analysis of the studies in which social anxiety-like behaviour was measured found no statistically significant difference in sociability between isolated and control animals, regardless of sex (*p* > 0.1, Fig. 1B). Substantial heterogeneity (*I*^*2*^ > 40%) between the studies was apparent throughout all meta-analyses. In summary, social isolation caused a moderate increase in generalised anxiety-like behaviour in male animals, while the effects on generalised anxiety-like behaviour in females and social anxiety-like behaviour in males and females were inconclusive, owning to substantial heterogeneity between studies.

**Fig. 2:**
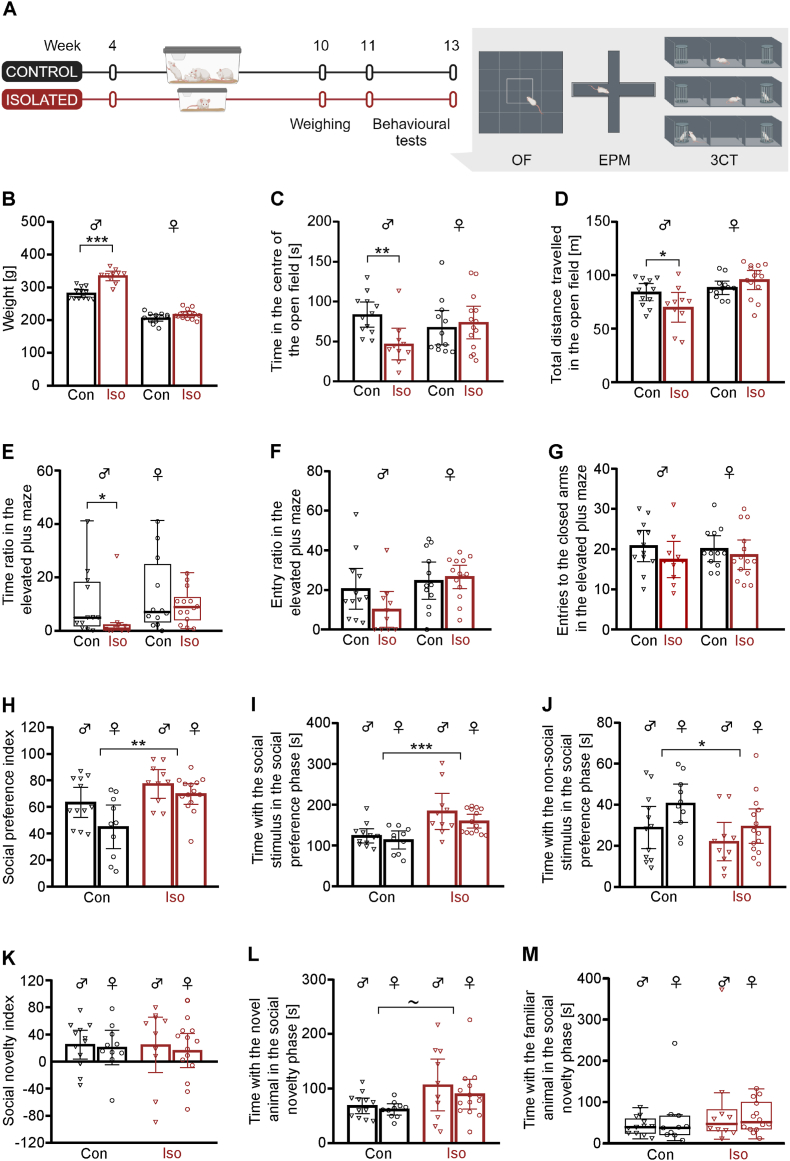
**Effect of social isolation during adolescence on weight, generalised and social anxiety-like behaviour in rats.** (A) Experimental design. (B) Weight measured after six weeks of social isolation was 19% (95%CI: 13–25%, *p* < 0.001) increased in male isolated rats, whereas there was no significant increase in female isolated rats (*p* = 0.11). (C) Male isolated rats showed 44% (95%CI: 18–70%, *p* = 0,008) decrease in time in the centre and (D) 17% (95%CI: 21–34%], *p* = 0.028) decrease in total distance travelled in open field, whereas females did not exhibit these decreases (*p* > 0.1). (E) Relative time spent in the open arms of the elevated plus maze was decreased (*p* = 0.034) in the isolated male animals (median: 0.76, IQR: 0.00–2.06) compared to control male animals (median: 4.96, IQR: 2.04–17.75), but not in female animals (*p* > 0.1). There were no statistically significant differences observed in (F) relative number of entries to the closed arms (*p* = 0.29) and (G) total number of entries to the closed arms (*p* = 0.17) in elevated plus maze test between isolated and control rats either sex. (H) Male and female isolated rats exhibited a 32% (95%CI: 13–51%, *p* = 0.001) increase in social preference index, (I) a 42% (95%CI: 24–61%, p < 0.0001) increase in time spent with social stimulus, and (J) 23% (95%CI: 3-49%, *p* = 0.046) decrease in time spent with non-social stimulus in social preference phase of three-chamber test. Male and female isolated rats also exhibited (L) a 48% (95%CI: 12–84%, *p* = 0.079) trend towards an increase in time spent with novel animal in social novelty phase of three-chamber test, whereas there were no significant differences observed in (K) social novelty index (*p* > 0.1) and (M) time spent with familiar animal (*p* > 0.1) in social novelty phase in three chamber test. Results are presented as dot-plots with annotated means ± 95%CI or as Tukey box-plot. Statistical annotations: ∼*p* < 0.1 *p < 0.05, **p < 0.01, ***p < 0.001.

To investigate the impact of chronic adolescence social isolation of rats on generalised and social anxiety-like behaviour, animals were randomised according to the litter and weight into single caged and group caged experimental groups at four weeks of age. Subsequently the animals were weighed and subjected to open field, elevated plus maze, and three-chamber social preference tests. Body weight was increased in the male isolated rats by 19% compared to control male rats, at tenth post-natal week, after six weeks of social isolation, whereas no significant change in body weight was observed in female isolated rats (Fig. 2B). The open field and elevated plus maze tests were used to investigate the generalised anxiety-like behaviour in rats after social isolation stress. In the open field test, isolated male rats exhibited increased generalised anxiety-like behaviour and decreased locomotion compared to control male rats, as measured by a 44% decrease in time spent in the centre of the open field (Fig. 2C) and a 17% decrease in total distance travelled (Fig. 2D), whereas no statistically significant difference was found between isolated and control female animals. In the elevated plus maze test, relative time spent in the open arms was also significantly decreased exclusively in male isolated rats indicating an increase in generalised anxiety-like behaviour (Fig. 2E). No statistically significant difference in the relative number of entries to open arms (Fig. 2F) or the total number of entries to closed arms in the elevated plus maze test was observed between isolated and control animals, regardless of sex (Fig. 2G). To investigate social anxiety-like behaviour after social isolation during adolescence, rats were subjected to the three-chamber test. Isolated rats of both sexes exhibited an increase in sociability, as measured by a 32% increase in social preference index (Figs. 2H), 42% increased time spent with social stimuli (Figs. 2I) and 23% decrease in time spent with non-social stimulus (Fig. 2J). Both male and female isolated rats also showed a trend towards an increased preference for novel social stimuli, as measured by a 48% increase in time spent with a novel animal during the social novelty phase (Fig. 2L). No statistically significant difference in social novelty index (Fig. 2K) or time spent with familiar animal (Fig. 2M) in social novelty phase in three-chamber test was observed between isolated and control rats. In summary, social isolation stress during adolescence causes increased generalised anxiety-like behaviour exclusively in male rats, while it leads to increased sociability in both male and female rats.

## 4 Discussion

The results obtained from both the retrospective analysis and the prospective experiment indicate that social isolation during adolescence caused generalised anxiety-like behaviour exclusively in male, but not in female rats. The retrospective analysis of previously published data indicates that the effects of social isolation on social anxiety-like behaviour is ambiguous, whereas the results of the current prospective experiment indicate an increase in sociability and consequently a decrease in social anxiety-like behaviour, which is contrary to expectations.

The considerable variability in results observed in the retrospective analysis within and between studies that focused on the stress model of social isolation in adolescents suggests that further research is needed to establish a representative animal model of social isolation in adolescence. However, the results of the behavioural experiment on social isolation in adolescence in male rats confirm the presence of an increase in generalised anxiety-like behaviour from the meta-analysis based on the literature review of similar studies. Moreover, the absence of generalised anxiety-like behaviour in females after social isolation in adolescence was also a finding derived from both the retrospective data analysis and the behavioural experiment. This result suggests that isolating female rats for a period of six weeks does not impose any additional stress on the animals in this behavioural domain. Thus, it can be concluded with reasonable certainty that the social isolation paradigm is a reliable method to elicit generalised anxiety-like behaviour in males. This model may be useful for prospective preclinical screening of anxiolytic drug candidates and for more in-depth studies focusing on the molecular mechanisms of anxiety elicited by social stressors; however, such studies would likely need to be conducted exclusively in males, and the experimental groups would likely need to include a substantial number of animals to account for bio-variability. It remains unclear whether the apparent sex difference in generalised anxiety-like behaviour triggered by social isolation stress is an inherent feature or a consequence of the methods used, which do not take the oestrus cycle into account. Therefore, it is essential for future research involving both sexes to investigate the effects of the oestrus cycle in females and to explore the underlying molecular mechanisms, in order to identify these apparent differences. The observed results appear to be very consistent among the reports, which emphasises the importance of understanding sex differences and adhering to the guidelines for conducting experiments on animal models of psychiatric disorders in both male and female rodents.

Regarding the effects of adolescence social stress on social anxiety-like behaviour, although the meta-analysis did not yield clear conclusions, it is evident that there are studies indicating an increase, no change, and a decrease, and that the outcome likely depends on numerous factors. Theoretically, the lack of social interaction during adolescence is thought to lead to a lack of experience with social contact and a potential aversion to such contact, which would translate into social aversion and social anxiety-like behaviour. On the other hand, sociability is an ingrained animal instinct that is not easily abolished in rodents; therefore, an instinctive desire for social contact may also lead to an adaptive response and increased sociability in socially isolated animals, even if the acquisition of social skills was impaired during adolescence. It is reasonable to assume that the former phenomenon occurred in some of the earlier studies, while the latter phenomenon occurred in the experiment presented here and in some earlier experiments.

## Declaration of competing interest

The authors declare no conflict of interest related to this manuscript.
